# A Bottom Up Perspective to Understanding the Dynamics of Team Roles in Mission Critical Teams

**DOI:** 10.3389/fpsyg.2019.01322

**Published:** 2019-06-11

**Authors:** C. Shawn Burke, Eleni Georganta, Shannon Marlow

**Affiliations:** ^1^The Institute for Simulation and Training, University of Central Florida, Orlando, FL, United States; ^2^Department of Psychology, Ludwig-Maximilians-Universität München, Munich, Germany; ^3^Department of Management, The University of Texas at San Antonio, San Antonio, TX, United States

**Keywords:** teams and groups, team roles, team performance, time, temporal and contextual factors

## Abstract

There is a long history, dating back to the 50 s, which examines the manner in which team roles contribute to effective team performance. However, much of this work has been built on *ad hoc* teams working together for short periods of time under conditions of minimal stress. Additionally, research has been conducted with little attention paid to the importance of temporal factors, despite repeated calls for the importance of considering time in team research (e.g., Mohammed et al., 2009). To begin to understand team roles and how temporal aspects may impact the types of team roles employed when teams are working in extreme mission critical environments, the current manuscript uses a data-driven, bottom-up approach. Specifically, we employ the use of retrospective historical data as our input and a historiometric approach (Simonton, 2003). Source documents consist primarily of autobiographies, memoires, biographies, and first-hand accounts of crew interaction during spaceflight. Critical incidents regarding team interaction were extracted from these source documents and independently coded for team roles by two trained raters. Results of the study speak to the importance of task and social roles within teams that are predominantly intact and operating in extreme environments where mistakes can be life threatening. Evidence for the following task (i.e., coordinator, boundary spanner, team leader, evaluator, critic, information provider, team player, and innovator) and social roles (i.e., team builder, nurturer, harmonizer, entertainer, jokester, and the negative roles of attention seeker and negativist) were found. While it is often task roles that receive the greatest attention, results point to the importance of not neglecting the socioemotional health of the team (and the corresponding roles). Results also indicated that while some roles were consistently enacted independent of temporal considerations (e.g., mission length), the degree to which others were enacted varied across missions of differing lengths. Additionally, based on the current sample we see the following trends: (1) increased enactment of the team builder role as mission duration increases, (2) prominence of the entertainer role, and (3) increased emphasis on the visionary/problem solver role on missions over 2 years.

## Introduction

It has often been said that a team of experts does not make an expert team. Although different conceptualizations of teams have been introduced within the literature, one prevalent definition stipulates that teams consist of two or more individuals who interact dynamically, adaptively, and interdependently; share common goals or purposes; and have specific roles or functions to perform (Salas et al., 1992). Teams represent a prevalent approach to structuring work, with a majority of employees reporting spending at least some part of their day within a team setting (Ken Blanchard Companies, 2006). In this vein, there is a long history of research that has sought to examine the factors that contribute to team effectiveness within a variety of contexts and much has been learned (Mathieu et al., 2008).

Despite the long history of research on team effectiveness, much of this work has been built on *ad hoc* teams working together for short periods of time within laboratory or organizational settings. Additionally, much of this work is primarily static in nature despite repeated arguments for the importance of considering temporal factors in team research (e.g., Mohammed et al., 2009). This, in turn, has led to minimal guidance for those individuals tasked with staffing, developing, and assessing teams that operate over longer periods of time as intact teams or operate within mission critical, extreme contexts. Teams that operate in these environments are often referred to as “extreme teams.” According to Bell et al. (2018), extreme teams are those which are embedded in environments whereby one or more contextual features exist that are atypical in level or kind.

While understanding the factors that facilitate team effectiveness and how these may change over time is an important and difficult endeavor due to the complexity of collecting longitudinal data on teams, facilitating this understanding is of even greater importance for teams operating in extreme contexts. Extreme teams are not only exposed to stressors that are atypical in level, but stressors often occur simultaneously and oscillate between chronic and acute duration levels (Bell et al., 2018). Teams operating under these conditions have been shown to be more likely to have decrements in performance due to the effects of stress on team process (and correspondingly performance, Driskell et al., 1999).

In seeking to understand the factors that facilitate the effectiveness of such teams and how these factors may change based on temporal factors (e.g., team duration), we focus on team roles. Research on team roles has a rich history dating back to Bales (1950). Roles have been defined as a “set of behaviors that are interrelated with the repetitive activities of others and characteristic of the person in a particular setting” (Stewart et al., 2005, p. 344). Throughout the years, many taxonomies have been created to delineate the roles that facilitate performance in teams (e.g., Bales, 1950; Belbin, 1981; Mumford et al., 2006). While there are differences in the taxonomies created throughout the years, nearly all argue for the importance of both task and social roles. However, not much is known regarding the types of team roles needed within mission critical, extreme contexts, or how team roles in this context vary based on temporal factors (e.g., team/mission duration).

Therefore, the goal of the current study is to move the literature forward in two thrusts: (1) understanding the team roles needed within extreme environments and (2) examining how the instrumentality of specific team roles may vary based on temporal factors in extreme environments. These advancements meet a critical need in better understanding the dynamic nature of teams and consequently the roles that are enacted, but also begin to highlight the importance of context.

To achieve our goals, we employ historiometry (Simonton, 2003) as a methodology to analyze archival documentation of crew interaction, with a particular emphasis on role enactment in extreme teams using spaceflight crews as an exemplar. In the following, we first present background on team roles, extreme teams, and highlight a set of hypotheses that serve to drive our approach. Next, we summarize our methodology including the nature of our sample and procedure. Finally, we describe our results, extract the implications for understanding the dynamic nature of team roles within the context of extreme teams, and highlight future research needs.

## Team Roles

Team roles have been defined as different functions and responsibilities team members must assume to enable smooth team functioning (Stewart et al., 1999, 2005). In this vein, a number of taxonomies have been created that argue for those roles that must be enacted to facilitate team performance (Benne and Sheats, 1948; Belbin, 1993; Mathieu et al., 2015; Driskell et al., 2017). The manner in which taxonomies have described team roles has varied, ranging from descriptions involving: (1) high overarching categories consisting of 2–3 dimensions, (2) nuanced categories consisting of 5–12 dimensions, and (3) those focusing on a set of core characteristics (see Table 1 for exemplars). Early work tended to describe team roles primarily in terms of broad overarching roles (e.g., Bales, 1950). Evidence of this research stream can still be seen in work on team roles for despite many role taxonomies becoming more nuanced, there is now general agreement on two broad classes of team roles: task roles (those behaviors that further task completion and fulfillment of the team’s objectives) and social roles (those behaviors that maintain the team’s social environment and the socioemotional health of the team).

**Table 1:** Example team role taxonomies.

Source	Description	Dimensions
Bales, 1950	Task roles Socioemotional roles	• Asking for/giving orientation, opinion, suggestions • Positive – show solidarity, tension release, agrees, negative – antagonism, tension, disagrees

Benne and Sheats, 1948	Task roles Group building/ maintenance roles Individual roles	• Initiator-contributor, information seeker, opinion seeker, information giver, opinion giver, elaborator, coordinator, orienter, evaluator-critic, energizer, procedural technician, recorder • Encourager, harmonizer, compromiser, gate-keeper, standard setter, group-observer, follower • Aggressor, blocker, recognition-seeker, self-confessor, playboy, dominator, help-seeker, special interest pleader

Belbin, 1993	No specification	• Completer-finisher, implementer, specialist, monitor-evaluator, coordinator, plant, shaper, resource investigator • Team worker

Mumford et al., 2006	Task roles Social roles	• Contractor (organize, coordinate), creator (promote innovative approaches), contributor (provides pertinent information), completer (foster task completion) critic (promote open discussion of potential issues) • Communicator (promote healthy social environment/collaboration), cooperator (conforms to others expectations), calibrator (observe/change team social processes), consul (gather information from outside sources), coordinator (coordinates team efforts with outside)

Mathieu et al., 2015	Task roles Socio-emotional roles Change-orientated roles	• Organizer, doer, challenger, innovator • Team builder, connector • Challenger, innovator

Driskell et al., 2017	Focus on dimensions which underly all roles in varying degrees	• Dominance • Sociability • Task orientation

As the literature progressed, taxonomies began to become more nuanced, accounting for a more varied set of roles (e.g., Margerison and McCann, 1985; Belbin, 1993; Parker, 1994, 1996; DuBrin, 1995). Perhaps most recent in this steam of work are role taxonomies put forth by Mumford et al. (2006) and Mathieu et al. (2015). Mumford et al. (2006) synthesized the previous literature on roles and delineated a set of ten roles, five task roles (i.e., contractor, creator, contributor, completer, critic) and five social roles (i.e., communicator, cooperator, calibrator, consul, coordinator, see Table 1). Mathieu et al. (2015) suggest that one of the key theoretical contributions of this work is integrating Ancona and Caldwell’s (1988, 1992) work on roles with additional theoretical frameworks to include the notion of boundary spanning. Work by Mathieu et al. (2015) attempted to find a middle ground between high overarching taxonomies of team roles and those taxonomies with many nuanced team roles. Mathieu et al. (2015) proposed and validated the Team Role Experience and Orientation (TREO), that includes six team roles. The six roles consist of the organizer (i.e., structures the team and task to ensure goals are being met), doer (i.e., completes taskwork), challenger (i.e., challenges the team to question assumptions and approaches to the task), innovator (i.e., generates ideas and solutions), team builder (i.e., maintains a positive atmosphere within the team, establishes norms, and supports team decisions), and connector (i.e., connects the team with outside entities). Taken as a whole, the research provides compelling evidence to support the validity of the six roles introduced within this theoretical framework.

Representing the last category of role taxonomies is the work of Driskell et al. (2017). Building upon previous work, Driskell et al. (2017) delve deeper into roles and argue that there are three characteristics (i.e., dominance, sociability, task orientation, see Table 1) that can be used to describe all team roles based on the degree to which each characteristic is present. This three-dimensional model is labeled TRIAD or Tracking Roles in and Across Domains. Its usefulness lies in helping to understand how team roles might covary with one another based on their underlying characteristics.

Each of these approaches has expanded an understanding of the team roles needed for successful teamwork. However, there remains a gap in the literature regarding the influence of context. Researchers have sought to create team role taxonomies that are comprehensive and generalize across samples and conditions. Yet, we suggest that the prevalence and necessity of team roles may be contingent upon the demands of the situation. Therefore, we draw from a taxonomy introduced to describe team roles in extreme environments to further understanding in this area. In particular, Burke et al. (2016) developed a taxonomy which utilized existing literature and interviews with domain experts to form an initial set of team roles grounded in the context of teams operating in extreme environments. The taxonomy depicts a set of eleven roles consisting of five social roles (three functional, two dysfunctional) and six functional task roles. Social roles include: contribution seeker, team builder, jokester/entertainer, attention seeker, and negativist. In contrast, task roles consist of the following: team player, evaluator, information provider, boundary spanner, visionary/innovator, coordinator (see Table 2 for a full description of roles).

**Table 2:** Team role taxonomy (Burke et al., 2016).

Team role	Description
**Social roles**	
Contribution seeker	Behaviors that seek to ensure that all members are contributing to the task, are recognized for their contribution, and feel their contribution is valued.
Team builder	Behaviors that seek to improve and maintain the social structure, motivation, and team well-being. This includes sub-roles: harmonizer, motivator, and nurturer.
Entertainer	Behaviors which serve to maintain cohesion and emotional well-being through humor and other active public forms of artistic expression targeted at the team. Subdimension: jokester.
Attention seeker	Behaviors that serve to consistently call attention to oneself. This attention seeking is self-initiated.
Negativist	Behaviors which reflect an explicit negative outlook, are toxic in nature, and serve to degrade the social emotional environment within the team. This includes sub-roles: complainer and aggressive arguer.
**Task roles**	
Team player	Behaviors which reflect a willingness to pitch in wherever is needed and being prepared to help. This includes sub-roles: task completer, mission support, and social loafer (negative instance).
Evaluator	Behaviors aimed at questioning and ensuring the best use of team ideas and information. This includes sub-roles: critic and analyzer/synthesizer.
Information provider	Behaviors which serve to transmit information within the team serving to create shared mental models. This includes the sub-roles of clarifier, facilitator, note taker, power seeker (negative role).
Boundary spanner	Behaviors which represent someone who is managing the relationship of the team with outside entities as well as gathering/sending information outside the team to bring back in.
Visionary/innovator	Behaviors which are oriented toward coming up with new and creative ideas and approaches to the task.
Coordinator	Leadership-oriented behaviors focused on the processes involved in task completion. The includes sub-roles: team leader, project manager.

While the taxonomy put forth by Burke et al. (2016) provides initial input into the types of team roles that may appear, further research needs to be conducted to examine the degree to which these roles actually occur in teams operating in extreme contexts. Teams embedded within extreme environments are repeatedly faced with strong situations which present unique demands, and each demand may require a different team role. Consequently, a more precise theoretical model explicating the roles needed for success, depending upon the various demands of the situation, is required. To address this gap, we leverage the taxonomy described by Burke et al. (2016) along with the literature on extreme teams (below) to foster our understanding of how different conditions faced by spaceflight teams influence the necessity of specific team roles.

## Role Enactment in Extreme Teams

As the predominant amount of work on team roles has been conducted within the context of teams operating in non-extreme environments, those charged with composing, managing, or developing teams that operate in extreme environments have little guidance upon which to rely; this is despite the mission critical nature of the teams that operate within these types of environments. Extreme environments have been described as ones in which “one or more extreme events are occurring or are likely to occur that may exceed the organization’s capacity to prevent and result in an extensive and intolerable magnitude of physical, psychological, or material consequences to – or in close physical or psycho-social proximity to – organization members” (Hannah et al., 2009, p. 898). Teams that operate within extreme environments often face stressors that are atypical in kind or level (Bell et al., 2018); this culmination of stressors may drive the instrumentality of the various task and social roles that have been argued for within the broader literature.

While there are a number of team types that operate in extreme environments, perhaps the most commonly referenced are those operating within the context of polar exploration, firefighting, spaceflight, and some military environments. In investigating role enactment within these more extreme teams, we utilize teams involved in space exploration/spaceflight. Teams operating within the context of spaceflight face a number of potential stressors that are atypical in terms of kind and level. For example, research has identified at least four different classes of stressors often present in this environment: physiological/physical, habitability, taskwork, and psychosocial (see Dietz et al., 2017). In terms of physiological/physical stressors the following have been identified: decreased exposure to sunlight, circadian rhythm disruption, and sleep deprivation. Stressors related to habitability have been argued to include things such as a lack of privacy, noise/vibrations, and cooking/eating restrictions. Crews also face task related stressors such as: scheduling, variations in task autonomy, periods of monotomy/boredom, shiftwork, time pressure, and high workload. Finally, there are a myriad of psychosocial stressors which may occur, including but not limited to family life disruption, multicultural issues, task and relationship conflict, communication delays, and isolation/confinement (Dietz et al., 2017). These stressors often occur in conjunction with one another and serve as a source of threat to the crews embedded within this environment. As such, space exploration, and the teams therein, provide an exemplar of teams that operate in extreme environments and can be categorized along the set of characteristics argued by Hannah et al. (2009) to define extreme environments (i.e., location in time, magnitude of consequences, probability of consequences, physical/psychosocial proximity, and form of threat).

In seeking to understand the team roles that must be enacted within extreme environments, such as spaceflight, we can leverage work conducted on how teams respond when under stress. In this vein, early work by Sorokin (1943) found that groups involved in catastrophic events tended to become overly aroused and emotional which consequently impacted the way they processed information and made decisions. Similarly, work conducted by Driskell and Salas (1991) found stress impacts the degree to which members are receptive to informaton offered by team members. Specifically, replicating previous findings (Foushee and Helmreich, 1988), Driskell and Salas (1991) found that under stress low status members became more willing to defer to high status members. However, contrary to previous findings, results indicated that high status members were more likely to attend to the task contributions of others. In these cases the team is in a situation in which the high status member is willing to accept task input, yet lower status members may be less willing to provide such input. This drives a need for task related roles which seek to proactively elicit information from relevant team members. While this role primarily serves to facilitate task accomplishment, it does have a social component by providing a sense of meaning and value to team members indicating that their contributions are valued.

Extending this work are findings by researchers indicating that stress leads to a loss of team perspective whereby an individual member’s breadth of attention narrows and they become more self-focused, less group identity is reported, and members have less of a collective representation of the task (Driskell et al., 1999). Similarly, stress has been argued to increase distraction and decrease attentional focus, increase team members’ cognitive load, increase negative emotion (e.g., frustration, fear, anxiety), and increase social impairment (e.g., reduce back-up behavior, increased interpersonal conflict/aggression, failure to appropriately read social cues, and less cooperative behavior as seen through attentional narrowing) (Driskell et al., 2018). Given the impact that stress has on both task and psychosocial aspects of the team, in line with prior research, we would expect that both task and social roles would be present (Prichard and Stanton, 1999; Chong, 2007) and fairly equally distributed when looked at across the lifecycle of the team.

Hypothesis 1: The distribution of task and social roles will be fairly equally represented in extreme teams.

The taxonomy put forth by Hannah et al. (2009) along with the types of stressors often experienced within spaceflight can be used to further make predictions regarding the specific types of task and social role behaviors that might be evidenced. Hannah et al. (2009) delineates five dimensions of extreme environments: location in time/temporal ordering, magnitude of consequences, probability of consequences, form of threat, and physical or psychosocial proximity. For the current effort, the first four of these are perhaps the most relevant in delineating the types of roles needed within the context of spaceflight (and other teams operating in similar extreme contexts). As such, these will be briefly discussed next.

### Location in Time

The types of threat that are present within the predominant number of extreme environments are ones which oscillate over time (e.g., at certain times being more of a concern). The temporal cycle of the impact of such threats will vary across extreme contexts and as such will drive the nature of the type of team processes required for teams to be resilient within such environments. With regard to spaceflight, the threat is primarily located in the situation although some physiological effects can persist beyond the immediate situation. While there are always low intensity chronic stressors that exist within spaceflight due to the mission criticality of the environment and distance of the crew from earth, there are periods of high intensity, acute stressors which may occur in combination as unexpected or off-nominal events occur. In this vein, Hannah et al. (2009) argue for the importance of the management of transitions between these periods of nominal and off-nominal events. With regard to roles, this drives the need for the sets of behavioral activities which will facilitate team and leader transition phase behaviors as seen in the work of Marks et al. (2001) and Morgeson et al. (2010). More specifically, role behaviors that facilitate structuring and planning of coordinative activities and points of transition, such that member cognitive and behavioral capacities are taken into account in order to ensure the capacity of any one individual member is not exceeded. This would, in turn, point to the importance of the coordinator role, information provider which serves to facilitate the exchange and clarification of information, boundary spanner to push and pull information in from outside the immediate team for use in planning, as well as the enactment of the evaluator role.

### Magnitude/Probability of Consequences

The second and third factors that Hannah et al. (2009) argue as defining characteristics of extreme environments are the magnitude and probability of consequences. With respect to spaceflight, the magnitude and probability of consequences is high given the distance from earth, relative isolation, and the environmental characteristics of space. To better understand the impact on the crew and the roles that may be important, we leverage existing literature on the impact of stress on teams along with that on high reliability organizations. Extracting from the literature on stress and teams, stress has been shown to degrade team process by causing: a narrowing of attention, loss of team perspective, degradations in coordination, and tendency for groupthink with low status members more willing to defer to others and less likely to speak up (e.g., Janis, 1972; Callaway et al., 1985; Driskell and Salas, 1991; Burke et al., 2008; Ellis and Pearsall, 2011). This points to team roles such as the critic (to combat groupthink) and boundary spanner (to bring in alternative information from outside and serving to combat the narrowing of attention and in combination with the critic role serving to combat groupthink). The propensity for low status members to “go with the flow” and potentially not offer valuable information drives the need for the contribution seeker.

High reliability organizations (HROs) can be defined as organizations that operate within environments where the magnitude and probability of consequence of error is high, yet are able to minimize errors (Roberts, 1990). As such, HROs should provide some insight into the types of roles needed when magnitude and probability of consequence is high. Research has suggested that principles of collective mindfulness (i.e., preoccupation with failure, reluctance to simplify interpretations, sensitivity to operations, commitment to resilience, and underspecification of structures, Weick et al., 1999) are the mechanisms that allow HROs to effectively operate. Moreover, work has attempted to translate the above organizational practices to the team level (e.g., Wilson et al., 2005; Baker et al., 2006). Wilson et al. (2005) argue that at the team level, these processes may be manifested through the following actions: sensitivity to operations (e.g., cross-lagged communication, information exchange, maintaining shared situation awareness), commitment to resilience (e.g., backup/monitoring, shared mental models), deference to expertise (e.g., assertiveness, collective orientation, expertise), reluctance to simplify (e.g., adaptability, flexibility, and planning), and preoccupation with failure (e.g., error management, feedback/team self-correction).

An examination of the HRO principles can provide insight into the types of team roles needed. For example, many of the principles speak to ensuring that information is being transmitted throughout the team (i.e., sensitivity to operations, preoccupation with failure) to maintain shared mental models and situation awareness (i.e., sensitivity to operations, commitment to resilience). This speaks to the need for team roles such as the information provider and contribution seeker to ensure relevant input is being gained no matter the status of the individual team member. The importance of members backing one another up (i.e., commitment to resilience) and maintaining a collective orientation (i.e., deference to expertise) drives the need for the team player, jumping in wherever needed. Finally, the requirement to be adaptive and flexible (i.e., reluctance to simplify, preoccupation with failure) drives the need of the critic who can combat against groupthink as well as the boundary spanner role to ensure that the team is maintaining an awareness of events outside the team that may impact their mission.

Hypothesis 2: The oscillations in stressor onset as well as the high magnitude and probability of consequences will drive the following task-orientated roles as being commonly seen: boundary spanner, team player, evaluator/critic, contribution seeker, and information provider.

### Form of Threat

The fourth characteristic along which extreme environments can be characterized is the form of the threat(s) presented to the teams. Hannah et al. (2009) argue that threats can be physical, psychological, or material. In the case of spaceflight, while threats can exist on any of the three aforementioned dimensions, they are most often physical and psychological. Factors such as isolation, confinement, and disruption of family life drive the increased need for team roles that are targeted at maintaining the psychosocial health of the team, in addition to the physical health. Therefore, we predict that the enactment of behavioral sets of activities that serve to reduce interpersonal conflict (e.g., harmonizer), maintain team morale, redirect crew attention from the negative aspects (e.g., team builder, entertainer), and ensure that personal physical and space needs are met (e.g., nurturer) are the key social roles that will be seen within extreme environments. The latter set of roles (e.g., nurturer) arise to fulfill the gap created based on the confinement and isolation from loved ones who might otherwise ensure these basic needs are met.

Hypothesis 3: Social roles that will be most prominently seen in extreme teams (e.g., spaceflight crews) include: the harmonizer, nurturer, team builder, and entertainer.

## Role Enactment and Temporal Considerations

While the contextual nature of extreme teams is expected to drive the importance and/or frequency of enactment of particular roles as argued for above, it is also expected that team roles are dynamic and the degree to which specific roles are manifested within a team will vary based on several temporal factors. Below, we begin to set forth a series of propositions driven by the literature on team development, albeit manifested in two different ways. The literature on team development and team dynamics has a long history (e.g., Tuckman, 1965; Gersick, 1991; Salas et al., 1992; Hackman and Wageman, 2005; Kozlowski et al., 2009; Burke et al., 2017), yet in thinking about extreme teams we take a slightly different approach in that we couple team development with contextual factors due to their tightly linked nature in teams.

The context within which we are investigating extreme teams is one in which the team members tend to be task experts, co-located with fellow crew members, and highly driven individuals. These crews also tend to be intact, operate under varied stressors that occur simultaneously, and tend to have high level of isolation and confinement. Therefore, our propositions will touch less upon the team developmental needs as by the time the predominant number of these teams are on a mission, they have already been exposed to a wide variety of team building and training exercises and in most cases have prior knowledge of crew members (if not prior working experience with them). Instead, we focus predominantly on how team needs may change over time based on the temporal duration of the missions within which the team is operating.

Work by Salas et al. (1992) has argued, and later research has shown (e.g., Mathieu et al., 2008), that in order to be effective, teams must master two tracks of skills – taskwork and teamwork. Specifically, the taskwork track represents “task-orientated skills that members must understand and acquire for task performance” (Salas et al., 1992, p. 10). In contrast, the teamwork track refers to “the behavioral interaction and attitudinal responses that team members must develop before they can function effectively as a team” (p. 11). We expect that teams operating in extreme contexts are no different than most operational teams in this regard (i.e., both sets must be mastered, as indicated by Hypothesis 1). However, we do propose that teams operating in these extreme environments have different challenges that cause the instrumentality of roles related to the maintenance of these two tracks to differ over time.

Within the set of extreme teams under consideration, missions of shorter duration tend to be characterized by high operational tempo due to the high workload present as crew members strive to complete science payloads, engage in public outreach and educational efforts, adhere to exercise and diet schedules, and ensure the equipment in transport vehicles and the habitat are working properly. The degree of high operational tempo seen in missions of short duration drives the crew into a very task-oriented mindset. Therefore, within these missions when the crew is together for shorter periods of time roles will tend to revolve around ensuring task needs are met. This is not to say that social roles are not important on the shorter duration missions, but the social stressors that the teams are exposed to on the short duration missions are not as salient as the task-orientated stressors. For these reasons, we would expect that in terms of frequency of enactment, there would be a greater proportion of task roles enacted on those missions that fall within the short duration category. The social stressors that the teams are presented with on short missions may be viewed as low level, while task stressors tend to be of higher levels and oscillate between acute and chronic in nature. Although not conducted with extreme teams, a review of team studies conducted by Bradley et al. (2003) revealed a pattern consistent with this expectation. They found that teams working on tasks of shorter duration, as compared to longer duration tasks, focused on “the task to the exclusion of efforts to form cohesive team norms that would only benefit the teams if they were going to remain together for the performance of future tasks” (p. 12). This evidence suggests that teams are less likely to invest in interpersonal relations and focus on fostering group norms via social roles when focused on tasks or missions of shorter duration (i.e., <=15 days).

Hypothesis 4: In shorter duration missions, task roles will be the driving factor in facilitating team performance, particularly those roles which foster the self-regulatory capacity of the team and facilitate collective mindfulness (e.g., boundary spanning, evaluator/critic).

As the duration of the mission increases, and correspondingly the team is exposed to the extreme conditions for longer periods of time, we expect that the enactment of social roles will become more prominent. The task-based stressors do not disappear as many are defining features of the extreme environment; however, the perceptions of isolation and confinement increase and begin to take a socio-emotional toll on the team. This effect is commonly reported in literature with respect to teams that have been deployed within extreme conditions for long periods of time. This phenomena is known as the third-quarter effect whereby individuals within isolated extreme environments often experience a decrease in mood and affect during the third quarter of their deployment or mission (Evans et al., 1987; Bechtel and Berning, 1991; Steel, 2001). This, in turn, is expected to drive an increased focus on behaviors that are related to ensuring that the social needs of the team are being met as a way to combat this natural drop in affect and mood. Moreover, teams formed for a longer period of time, as compared to teams working on tasks of shorter duration, have been found to invest more effort in forming relationships with other team members because they are aware that the longer task duration makes it more beneficial to have these relationships (Bradley et al., 2003). In line with this evidence, we suggest this is another reason, in addition to contending with the extreme environment (e.g., Steel, 2001), that more social roles are likely to be enacted on longer duration missions. Team members may engage in more social roles with the underlying goal of forming close relationships with other team members due to the longer duration of the mission.

Hypothesis 5: As team duration increases within extreme contexts the enactment of social roles become more frequent. Particularly, those roles that foster the socioemotional health of the team such as behaviors which provide an escape from the stressors present as well as behaviors which seek to maintain the emotional and physical health of the team (e.g., entertainer/jokester, nurturer).

## Materials and Methods

In order to test our assumptions and to gain a better understanding of team roles in extreme teams, with an emphasis on spaceflight crews, a historiometric approach (Simonton, 2003) was applied. Historiometry describes the systematic analysis of the content of past events and is defined as the “collection of methods in which archival data concerning historic individuals and events are subjected to quantitative analyses in order to test nomothetic hypotheses about human thought, feeling, and action” (Simonton, 1998, p. 269). This method is especially useful for exploring a relatively new research area, such as examining the dynamic nature of team roles in extreme environments, because it depends on data that were not explicitly collected for the research question of interest, thus limiting some bias. Further benefits of this approach include the contextual richness of the data and the corresponding external validity (Crayne and Hunter, 2018). Historiometry also enables the examination of complex constructs as expressed in behavior (e.g., team roles) during real situations, and the investigation of how such (team) constructs may differ depending on the type of situation (Antonakis et al., 2003). Recent studies have similarly applied historiometric analysis to explore topics such as team leadership in mission critical/isolated environments, successfully providing insight into other relatively new team-level research areas (e.g., DeChurch et al., 2011; Burke et al., 2018).

### Sample

The final sample used to examine our hypotheses consisted of 525 roles extracted from 514 critical incidents describing collective team interaction within the context of spaceflight. The incidents and coded roles came from the following seven missions that varied in length, allowing an examination of how team roles may vary over time: Shuttle, Soyuz, Gemini, Skylab, Salyut, Mir, and Mars 500.

### Procedure

#### Sources

The first step was to identify historical events (i.e., missions) that documented team interaction within the context of spaceflight. Sources were identified through the following databases: EBSCOhost, Google, and Google Scholar. Sources were also identified by searching the following websites: Amazon, Johnson Space Center, and European Space Agency. Both primary (e.g., diaries and autobiographies) and secondary sources (e.g., biographies and missions reports) (Simonton, 1990) were collected (see Table 3 for complete list of final sources used). Sources were examined for the extent to which they described team interaction and corresponding behaviors whereby critical incidents regarding team role enactment could be extracted. Of specific interest was task and social role enactment as evidenced within collaborative activities that occurred while members were engaged in their primary tasks (i.e., task execution) as well as those that occurred during off-task periods (i.e., downtime). Information related to duration of the spaceflight missions comprising our sample was also collected (Table 4). The missions identified fell into one of four durations: short (15 days or less), medium (greater than 15 days, maximum 6 months), long (greater than 6 months, maximum 1½ years), longest (longer than 1½ years, maximum 2 years).

**Table 3:** Sources and the respective spaceflight context.

Document title	Source	Author	Date	Spaceflight
				context
Flight: my life in mission control	Book (Autobiography)	Kraft C.	2001	Gemini
Of emergencies and Christmas trees – an exciting end to 2010	Diary (Mission Diary Entry)	Charles R.	12 January 2010	Mars 500
Goodbye Sun, goodbye Earth, we are leaving for Mars!	Diary (Mission Diary Entry)	Urbina D. and Charles R.	3 June 2010	Mars 500
Romain Charles completes the tour	Diary (Video Diary Entry)	Charles R.	21 June 2010	Mars 500
A dirty job but someone’s gotta do it!	Diary (Video Diary Entry)	Urbina D.	30 June 2010	Mars 500
This is our home, our workplace, and our life	Diary (Mission Diary Entry)	Urbina D.	7 July 2010	Mars 500
It’s housecleaning day	Diary (Video Diary Entry)	Urbina D.	14 July 2010	Mars 500
Smooth routine’ and interplanetary birthday party	Diary (Mission Diary Entry)	Charles R.	22 July 2010	Mars 500
Romain collecting air samples	Diary (Video Diary Entry)	Charles R.	9 August 2010	Mars 500
Waste not – want not	Diary (Mission Diary Entry)	Urbina D.	18 August 2010	Mars 500
How supplies are rationed?	Diary (Video Diary Entry)	Charles R.	6 September 2010	Mars 500
Science and thoughts of Chilean miners	Diary (Mission Diary Entry)	Charles R.	10 September 2010	Mars 500
Thanks to Oliver and Cyrille!	Diary (Mission Diary Entry)	Urbina D. and Charles R.	15 September 2010	Mars 500
Diego and Romain answer your questions	Diary (Mission Diary Entry)	Urbina D. and Charles R.	24 September 2010	Mars 500
Preparing the meals (with a shaker)	Diary (Video Diary Entry)	Urbina D.	12 October 2010	Mars 500
Diego and Romain answer your questions 2	Diary (Mission Diary Entry)	Urbina D. and Charles R.	26 October 2010	Mars 500
The barber shop on the way to Mars	Diary (Video Diary Entry)	Urbina D. and Charles R.	3 November 2010	Mars 500
“Are we alone?”	Diary (Mission Diary Entry)	Urbina D.	10 November 2010	Mars 500
Approaching the Red Planet	Diary (Mission Diary Entry)	Urbina D.	26 January 2011	Mars 500
Unpacking the Lander and preparing for a hike on Mars	Diary (Mission Diary Entry)	Charles R.	9 February 2011	Mars 500
Celebrating Chinese New Year – even on Mars!	Diary (Mission Diary Entry)	Charles R.	2 February 2011	Mars 500
Greetings from Mars!	Diary (Mission Diary Entry)	Urbina D.	1 March 2011	Mars 500
Long trip without moving anywhere	Diary (Mission Diary Entry)	Charles R.	3 June 2011	Mars 500
“The best moments of our trip”	Diary (Mission Diary Entry)	Charles R.	17 August 2011	Mars 500
Earth approaching!	Diary (Mission Diary Entry)	Urbina D.	13 October 2011	Mars 500
Countdown is on	Diary (Mission Diary Entry)	Charles R.	25 October 2011	Mars 500
Way station to the Stars: the Story of Mir, Michael, and Me	Book (Autobiography)	Foale C.	1999	Mir
Diary of a Cosmonaut: 211 days in Space	Book (Autobiography)	Lebedev V.	1990	Salyut
Salyut – The First Space Station: Triumph and Tragedy	Book	Ivanovich G. S.	2008	Salyut
Space Shuttle Columbia (Her Missions and Crews)	Book	Evans B.	2003	Shuttle
Riding Rockets: The Outrageous Tales of a Space Shuttle Astronaut	Book (Autobiography)	Mullane M.	2007	Shuttle
Space Shuttle Challenger (Ten Journeys Into the Unknown)	Book	Evans B.	2007	Shuttle
The All-American Boys: An Insider’s Candid Look at the Space Program and the Myth of the Super Hero	Book	Cunningham W.	2010	Shuttle
Homesteading Space: The Skylab Story	Book	Hitt D., Garriott O., Kerwin J., Bean A. L., and Hockam H.	2011	Shuttle
Wheels Stop: The Tragedies and Triumphs of the Space Shuttle Program, 1986–2011	Book	Houston R.	2014	Shuttle
Women in Space [Biography (Lerner Hardcover)]	Book	Gibson K. B.	2014	Shuttle
A House in Space	Book	Cooper H. S. F.	1976	Skylab
Around the World in 84 days: The Authorized Biography of Skylab Astronaut Jerry Carr	Book (Autobiography)	Shayler D. J.	2006	Skylab
Leaving Earth: Space Stations, Rival Superpowers, and the Quest for Interplanetary Travel	Book	Zimmerman R.	2003	Soyuz, Mir, and Salyut

**Table 4:** Differentiating of spaceflight context based on mission duration.

Duration	Exemplar missions	Incidents extracted
Short (<=15 days)	Shuttle, Gemini	132
Medium (<=6 months)	Skylab/ISS, Soyuz	124
Long (<=1.5 years)	Salyut, Mir	197
Longer (<=2 years)	Mars 500	72

#### Sampling

The initial search produced approximately 150 sources for further examination. Sources were then examined with respect to the following criteria: (a) sources must describe interdependent interaction among the crew/team; (b) sources must describe crew/team actions where team role behaviors (positive or negative) are present and described; (c) teams being described must be operating in a real or simulated spaceflight environment; and (d) source must be accessible. A group of psychologists with experience in team roles and historiometric analysis reviewed the suitability of all sources as described previously, while taking into consideration the representation of all different spaceflight contexts and missions durations. At the end of this stage, a set of 39 sources remained (i.e., 14 books and 25 diaries).

In order to systematically extract all relevant information from the final set of 39 sources, seven subject matter experts were trained on the critical incident technique and its application in the current context (Flanagan, 1954). The critical incident technique has been described as a set procedures that assist in the systematic extraction of human behavioral observation which may be “…adapted to meet the specific demands of the situation at hand” (p. 335). The first step in developing a critical incident is to understand the aim of the incident. For us, the aim is driven by our stated research questions. Therefore, the raters responsible for extraction of the critical incidents needed to understand what team roles were and how they manifest in teams. While all raters had a prior familiarity with team roles, ensuring their understanding was the initial part of our training. Next, training progressed to incident extraction. While the specific form a critical incident may take can vary based on the researcher’s need, for the current project, extraction included a behavioral description of team interaction at a specific point in time during the team’s mission as well as the consequence of that interaction (see Table 5 for examples).

**Table 5:** Example statements and categorization.

Sample critical incidents	Role	Source
“We did some funny TV today. Bill made some large cardboard swim fins, and paddles for his hands, and I televised him in his crazy get-up trying to paddle from one end of the forward compartment to the other. He put lightning bolts on the helmet. I laughed so much I could hardly hold the camera. I made up the dialog to go with it – called him “William Pogue Aerospace Pioneer.” Hope the folks on the ground get a kick out of it.”	Entertainer/jokester	Shayler, 2006
“Thus at an early stage Michael was able to show hospitality to his commander and flight engineer by welcoming them to his quarters to watch a late-night film, after supper together in the Base Block. They were glad of this entertainment and crammed amiably close to each other to watch Michael’s tiny movie theater. …In this way, almost by accident, he set up an early bond with his crewmates which presaged friendship and trust beyond anything normally required in the contracts or international agreements, or in previous binational crews’ experience. This warmth of feeling led to Michael’s first public support of his crewmates against their seemingly rather hard Ground Control taskmasters in Moscow.”	Team builder	Foale, 1999
Jean-Loup said, “I was surprised and impressed by your work together and how you fought to save the experiment.” He smiled and was also in a perfect mood.	Contribution seeker	Lebedev, 1990

“Carr complained that the soap was like dog shampoo. Pogue, the pilot, bitched that the towels–which were made of a synthetic material that was highly fire-resistant–were “sort of like drying off with padded steel wool.” Gibson griped that “the fire-prevention guys really got away with something when they made us go with that kind of material; I don’t think it’s absorbent enough, and I think it’s too hard.”	Negativist	Cooper, 1976
“Garriott, a bemused-looking, thin-faced man with a distinctive mustache that made him look like a western cowpoke, was even more eager to do more. Not only did he urge his crewmates on, he continually requested more work from scientists on the ground. ” “I was thrilled with my crew. Hoot Gibson was a natural-born leader. He didn’t micromanage as some commanders did (one was known to reach completely across the cockpit to make a switch change rather than allow the crewmember at that position to do it.) Hoot gave each of us our duties and set us free to be creative to get the job done.”	Team Player Coordinator	Zimmerman, 2003 Mullane, 2007
“This morning I suggested to Ground Control that we check the C-2 sextant and asked them to give us the location of three or four stars so that we could see one in the middle of the porthole.”	Boundary spanner	Lebedev, 1990

#### Coding

Once extracted, all incidents were double-coded by two SMEs with experience in teams (and more specifically team roles). The SMEs were asked to independently sort the identified roles into role type (i.e., social, task, or non-applicable), role category (e.g., team player, contribution seeker, or non-applicable), and if applicable into role subcategory. Raters utilized the Burke et al. (2016) taxonomy as a baseline for their coding, but were told not be restricted by the dimensions contained within that particular taxonomy. For some incidents, more than one role category was identified. For testing the interrater reliability among the SMEs, we calculated Krippendorff’s alpha, a standard reliability measure regardless of the number of observers, levels of measurement, sample sizes, and presence or absence of missing data, by using the respective SPSS macro (Hayes and Krippendorff, 2007). The interrater agreement was excellent for role type (Krippendorff’s α = 0.79), role category (Krippendorff’s α = 0.77), and for role subcategory (Krippendorff’s α = 0.75) (Cicchetti, 1994). In the final step, a meeting was held where both SMEs came to consensus regarding any discrepancies in their codes.

### Data Analysis

Data analysis consisted of two primary foci. First, to examine the set of propositions pertaining to team role enactment within extreme teams (Hypotheses 1–3), the roles that emerged from the card sort were rank-ordered by their frequency of occurrence. The frequency of each role type (i.e., task, social), role category (e.g., jokester, critic) and role subcategory (if applicable) was calculated.

To examine the dynamic nature of the identified team roles, we differentiated between spaceflight contexts in terms of the mission’s duration (i.e., short, medium, long, and longer duration, see Table 5). Specifically, we adopted a comparative method (e.g., Gardner, 1993) by comparing and contrasting the illustrated team roles, in order to extract the common and differing role characteristics between the various temporal durations.

## Results

### Team Roles

One of the primary questions posed within the current study was with regard to the types of task and social roles exhibited in teams operating within extreme contexts, using spaceflight crews as an exemplar. Closely related to this question was an examination of how temporal factors (i.e., mission duration) impact the nature of team roles exhibited. In this vein, five hypotheses were put forth regarding the team roles expected to be the most prevalent based on the defining features of spaceflight crews operating in extreme contexts and the frequency of specific role enactment based on mission duration.

With respect to Hypothesis 1, as predicted, results indicate that in terms of frequency both task and social roles were enacted in nearly equal proportions. Specifically, collapsing across missions, results indicated that 51% of the roles witnessed were social roles, while 49% of the roles were task-related (*N* = 267 and 258, respectively). Additionally, results indicated that many of the roles seen in previous taxonomies developed with respect to teams operating in more traditional, non-extreme environments also appeared in the current context (e.g., team builder, jokester, team player, information provider). However, at a global level there were some differences to note. First was the presence of the social role of “entertainer.” While similar to the jokester role seen in many role taxonomies outside of extreme contexts, the entertainer role is broader. Specifically, we define it as behaviors which seek to maintain cohesion and emotional well-being of team members through humor and other active, public forms of artistic expression. Additionally, the role of “nurturer” was a prominent role that does not often appear outside this context. This role consists of behaviors primarily focused on the maintenance of the physical health and personal space of crew members. Finally, of note is the lack of enactment of what would traditionally be considered negative roles consisting of behaviors directed at fellow team members (e.g., attention seeking, social loafing, expression of negativity). While a negativist role was frequently seen in some contexts it tended to consist of negative affect (i.e., complaining) regarding environmental, contextual, or equipment difficulties; it did not tend to be directed toward fellow crew members. When it was directed at individuals, it was most often members of ground control.

Hypotheses 2–3 described the task and social roles that were believed to be the most critical to teams operating in extreme contexts, such as spaceflight. To examine the data in relation to the hypothesis presented herein, the team roles that emerged from the card sort were rank-ordered in terms of their frequency of occurrence with respect to task and social roles, respectively. With respect to the predictions set forth in Hypothesis 2, findings were mixed. In line with predictions, the roles of boundary spanner, team player, and information provider emerged within the top five most frequently occuring task roles (see Table 6). The team player role is comprised of behaviors that reflect a willingness to pitch in wherever help is needed. Whereas, the information provider is comprised of behaviors serving to transmit and gather informaton within the team and create shared mental models. Finally, the boundary spanning role involves those behaviors which serve to maintain a link between the team and external entities and may involve the pulling and pushing of information. However, also occuring within the top five, but not predicted, were the coordinator role (encompassing subroles of team leader and project management) and the visionary/innovator role. The later role involving behaviors related to problem solving and thinking outside the box. Finally, contrary to predictions, behaviors related to the analysis and evaluation of ideas (e.g., critic) did not appear within the top five enacted task roles.

**Table 6:** Rank ordering of the top five task roles which emerged.

Team role	Rank order	% of comments
		supporting rank
Boundary spanner	1	55%
Team player	2	14%
Visionary/innovator	3	9%
Coordinator	4	5%
Information provider	5	4%

Hypothesis 3 pertained to the enactment of social roles. Similar to Hypothesis 2, results suggest partial support for this prediction. As expected, the team builder, entertainer, and nurturer roles were witnessed within the top five most enacted social roles (see Table 7). This reflects the importance of positive behaviors that improve the team‘s social structure and well-being. Specifically, the team builder reflects behaviors which seek to improve and maintain the social structure of the team, including behaviors that foster motivation and harmony. A subrole of this dimension is the nurturer role which primarily focuses on behaviors promoting the physical and emotional well-being of crew members, including personal space. However, the presence of behaviors reflecting an explicit negative outlook (i.e., the negativist) was unexpected. In further examining the results, these role behaviors primarily came from crews involved in the Skylab mission where relations between mission control and the crew degraded to such a point that the crew went on strike. Dropping the mission where the crew went on strike does drastically reduce the prevalence with which these behaviors are seen, but they would still appear within the top five. However, the focus then becomes negative comments related primarily to environmental and equipment conditions, with much less of a focus being on interpersonal negativity. Table 8 contains a full listing of all team roles which emerged and the frequency with which emergence took place (both task and social).

**Table 7:** Rank ordering of the top five social roles which emerged.

Team role	Rank order	% of comments
		supporting rank
Team builder	1	37%
Negativist	2	27%
Entertainer	3	26%
Nurturer	4	3%
Harmonizer	5	2%

**Table 8:** Relative frequency of enactment of task and social roles (as compared to one another).

	Across all temporal contexts	
Roles	*n*	%
Task roles	258	49
Team player	38	7
Task completer	15	3
Mission support	1	0
Evaluator	6	1
Analyzer/synthesizer	9	2
Information provider	10	2
Clarifier	5	1
Facilitator	1	0
Power seeker	1	0
Boundary spanner	111	22
Visionary/innovator	24	5
Coordinator	15	3
Team leader	17	3
Project manager	5	1
Social roles	267	51
Contribution seeker	3	1
Team builder	88	17
Harmonizer	8	2
Motivator	5	1
Nurturer	12	2
Entertainer	74	14
Attention seeker	8	2
Negativist	25	5
Belittler	–	–
Complainer	44	8

Additionally, we conducted exploratory analyses to determine the five most commonly enacted roles when looking across the total set of task and social roles. As can be seen in Tables 8, 9, results indicated the following five roles were the most frequently occurring, in order: boundary spanner, team builder, entertainer, negativist, and team player. This last role was closely followed by the presence of the visionary/innovator role. In essence this analysis pits social and task roles against one another to examine the most frequently occurring roles across the set of extreme contexts.

**Table 9:** Rank ordering of the top five team roles enacted across task and social categories.

Team role	Role type	Rank order	% of comments
			supporting rank
Boundary spanner	Task	1	22%
Team builder	Social	2	17%
Entertainer	Social	3	14%
Negativist	Social	4	8%
Team player	Task	5	7%

### Roles Over Time

Another primary goal of our study was to investigate the degree to which roles may vary across spaceflight contexts in terms of mission duration. As is common with the exploration of phenomena on which there is not a large body of prior work upon which to build hypotheses (and one reason for the approach taken), the hypotheses concerning the specific task and social roles expected to be most prevalent based on temporal duration received mixed support. Table 10 contains the full list of task and social team roles, their frequency counts and percentages as delineated by temporal duration.

**Table 10:** Emergence of team roles by temporal duration of mission^4^.

	Short duration		Medium duration		Long duration		Longer duration		Across contexts	
Roles	*n*	%	*n*	%	*n*	%	*n*	%	n	%
**Task roles**		
Team player	13	10	7	6	12	6	6	8	38	7
Task completer	12	9	–	–	–	–	3	4	15	3
Mission support	–	–	–	–	–	–	1	1	1	0
Evaluator	3	2	1	1	2	1	–	–	6	1
Analyzer/synthesizer	–	–	1	1	3	2	1	1	9	2
Information provider	3	2	1	1	3	2	3	4	10	2
Clarifier	2	2	2	2	1	1	–	–	5	1
Facilitator	–	–	–	–	–	–	1	1	1	0
Power seeker	–	–	–	–	1	1	–	–	1	0
Boundary spanner	15	11	24	19	67	34	5	7	111	22
Visionary/innovator	8	6	4	3	2	1	10	14	24	5
Coordinator	7	5	2	2	6	3	–	–	15	3
Team leader	13	10	1	1	1	1	2	3	17	3
Project manager	4	3	1	1	–	–	–	–	5	1
**Social roles**		
Contribution seeker	1	1			2	1			3	1
Team builder	7	5	22	18	32	16	27	38	88	17
Harmonizer	3	2	–	–	4	2	1	1	8	2
Motivator	2	2	1	1	2	1	–	–	5	1
Nurturer	5	4	1	1	5	3	1	1	12	2
Entertainer	18	14	18	15	30	15	8	11	74	14
Attention seeker	3	2	1	1	4	2	–	–	8	2
Negativist	3	2	8	7	14	7	–	–	25	5
Belittler	–	–	–	–	–	–	–	–	–	–
Complainer	6	5	29	23	6	3	3	4	44	8

*^*4*^Percentages contained in table are based on the total task and social roles enacted for a given mission.*

Results indicated that during short missions (i.e., less than 15 days), task team roles emerged twice as frequent (*N* = 84) as social roles (*N* = 48), while during medium duration missions (i.e., up to 6 months), the exact opposite role distribution was found between task (*N* = 44) and social (*N* = 80) team roles. During long (i.e., up to 1.5 years) and longer spaceflight missions (i.e., more than 2 years), the task (*N* = 98 in long missions, *N* = 32 in longer missions) and social (*N* = 99 in long missions, *N* = 40 in longer missions) team roles were evenly distributed. It seems that task roles are notably salient in very short missions, while social roles are gaining importance as the duration of the mission increases. At the same time, when the duration of the spaceflight missions exceeds a duration of 6 months both task and social team roles become equally frequent (see Table 11). The above set of results tends to support the primary tenets put forth in Hypotheses 4 and 5. Specifically, that the enactment of task roles are the most prominent within missions of short duration, while social roles gain more prominence as mission duration increases.

**Table 11:** Frequencies of task and social roles identified for each mission duration.

	Short duration		Medium duration		Long duration		Longer duration	
Roles	*n*	%	*n*	%	*n*	%	*n*	%
Task roles	84	64	44	36	98	50	32	44
Social roles	48	37	80	65	99	50	40	56

However, in looking at the predictions as to what particular task and social roles would appear most prominently, we received mixed results (see Table 10). One of the top task role categories, similarly frequent in all mission durations, was the team player, highlighting the importance of being willing and prepared to contribute and help whenever and wherever needed. The boundary spanner role also emerged as one of the top task roles in all mission durations, gaining frequence with increasing duration up to long duration missions; during the longer duration missions, the frequence of the boundary spanner was lower compared to the other mission durations. The opposite trend emerged for the third top task role for all mission durations – visionary/innovator; this social role decreased in frequency as mission duration was increasing, demonstrating its lowest frequency during long duration missions. For the longer mission duration, the visionary/innovator role emerged more frequently than in any other mission duration. The task role of team leader, highlighting the importance of leadership-oriented behaviors focusing on directing the teams toward mission completion, was identified as one of the top social roles only in short duration missions.

The entertainer role was one of the top social roles that similarly emerged in all mission durations, demonstrating the relevance of positive behaviors that serve to bring humor into the team. The team builder was identified as one more top team role in almost all spaceflight contexts, gaining frequence with increasing mission duration. During short duration missions, the frequency of the team builder role was noticeably lower compared to the other mission durations. The complainer team role, reflecting negative behaviors of complaining and whining about social team issues, emerged as one further top social role only for medium mission duration.

## Discussion

The use of teams has become ubiquitous within organizations due to the potential for teams to accomplish complex and interdependent work within environments that are increasingly dynamic. A well coordinated team is not only a pleasure to watch, but can bring tremendous rewards to organizations by leveraging the combined intellectual strength of its individual members. However, more often is the case that teams are implemented, yet fail to fully capitalize on the potential synergy present in the team; when capitalized upon, this synergy allows teams to become more than the sum of their individual member contributions. In effort to facilitate the probability that teams can leverage this potential capacity, there has been a tremendous amount of research conducted on the factors that facilitate the ability for members to work in a coordinated and adaptive manner such that they are ready to respond to changes both internal and external to the team. Due to the tremendous growth in team research and the corresponding lessons learned, a great deal of guidance can be currently provided to organizations regarding team dynamics. However, as noted by the editors of this special issue, sorely lacking in the area of team research is guidance pertaining to how the instrumentality of processes, states, and facilitating factors seen in team effectiveness models and team taxonomic efforts may vary due to temporal factors.

Due to the complexity of teams there are a variety of ways that temporal factors could be operationalized within teams, including but not limited to: the moment to moment changes in team process dynamics, oscillations between transition and action phase while engaging in a performance episode, team developmental stage, and/or length of time the team has been together. Within the current study, we have begun to take initial steps to delineate how team roles may vary over time by examining teams operating within extreme environments over short, medium, and long durations. Given our interest in team roles and how they may change over time within extreme teams, we chose to initially investigate this phenomena at a more global level in terms of time. The path we chose was dictated by the fact that, while dynamic, the enactment and switching of roles is most likely not as dynamic as changes in team process, thereby pushing us initially toward a more global view of time. In addition, given the lack of research on team roles over time within teams operating in extreme conditions we did not feel the theory was yet there to begin to predict moment to moment changes at a fine-grained level.

Results of the study speak to the importance of task and social roles within teams that are predominantly intact and operating in extreme environments where mistakes can be life threatening. Additionally, our findings begin to highlight areas of commonality and distinction between these environments and the more traditional organizational environments in which teams have been studied. In essence, while there were many commonalities between the team roles seen in the context of spaceflight and those which appear in the team role taxonomies which appear in the broader literature on teams, there were also differences. In terms of commonalities, task roles such as the team player, coordinator, evaluator/synthesizer, information provider/facilitator were seen. However, far less commonly seen were task related roles that may be considered dysfunctional (e.g., social loafer, power seeker). The decreased prevalence of these roles may be due to the mission critical environment in which the teams in this sample (and many teams in extreme environments) are embedded. Mistakes in these environments can often be extremely costly not only in terms of material, but personal resources – in some cases life threatening.

Many of the differences seen in terms of role enactment dealt with aspects of the social roles. Perhaps most prevalent was the expansion of the traditional jokester role to encompass a more inclusive entertainer role. This role reflects the elevation of mood and team member bonding not only through humor, but also through competitive activities and coming up with novel ways to occupy “down time.” Additionally, the team builder role incorporated the notion not only of behaviors which serve to reduce conflict and promote harmony among team members, but behaviors that serve to keep the team motivated, and behaviors that are more “nurturing” by nature. This later aspect of the team building role is one that is not often explicitly mentioned in the team taxonomies that appear in the broader literature. Finally, it is interesting to note that results did suggest a prevalence of behaviors related to negative affect; however, the predominant amount of these affective remarks were not directed at the immediate crew, but were either directed outside the immediate crew, or expressed in relation to conditions or equipment. This points to the fact that the atypical stressors present in the environment do serve to impact the affect of teams within extreme contexts; being resilient in these environments does not mean that negative affect does not occur. Future research should further investigate the mechanisms through which the team deals with the negativity when expressed. It is likely that some of the other social roles seen may serve as a buffer against the negativist comments, but this needs to be further investigated.

Furthermore, the exploration into how mission duration, or the degree of time that the team is embedded within the extreme environment, also revealed interesting findings. In particular, variation in the instrumentality of task role enactment on missions of shorter duration and the increased prevalence of social roles as mission duration increased. This points to the increased attention paid to the socioemotional impact that operating within extreme environments can have on the team and the types of social roles that teams utilize to mitigate some of these negative effects and remain resilient to the multitude of stressors. Often when examining teams operating in extreme environments there is a tendency to focus on the task-related effects of the stressors, with less of a focus on the socioemotional aspects. The findings from the current study begin to highlight the increased importance of not neglecting the socioemotional health of the team. Additionally, based on the current sample we see the following trends: (1) increased enactment of the team builder role, (2) prominence of the entertainer role, and (3) increased emphasis on the visionary/problem solver role on missions over 2 years. Of additional interest is the continued prevalence of the boundary spanner role even though these teams were operating under conditions of isolation and confinement. In part the prevalence of this role may be an artifact of the sample itself reflecting the communication between the flight crew and mission control. However, the role of boundary spanner has also been seen in extreme teams outside the context of spaceflight (Burke et al., 2018). Future research should continue to investigate the nature and instrumentality of this role under varying levels of isolation and confinement.

### Limitations

The examination of archival accounts of teams operating in extreme contexts provides a wealth of contextually rich information concerning real teams operating together over time. However, as with any method, it also has limitations. For example, it does not facilitate an understanding of the relationship of identified team roles to their impact on team processes and emergent states. Additionally, the source documents which were examined to pull critical incidents from were not written with our research questions in mind. While this may be considered a strength, as it may serve to eliminate biases concerning social desirability, given the archival nature it does not negate the possibility that the individual accounts themselves are biased. We attempted to mitigate this possibility to the extent it was possible by collecting information from multiple sources. Related to the fact that the source documents were not written for our specific purposes is the fact that while they were contextually rich they do not provide the level of detail needed in order to investigate team roles at a finer grained temporal level to capture more moment-to-moment changes. Future research should continue to explore these questions using a cross-section of methodologies as each method has its own strengths and weaknesses and it is only through a combination of methodologies that confidence will grow and theory will move forward.

### Future Research

The results herein begin to highlight those task and social roles that are important within extreme teams. While we did not explicitly compare high and low performing teams in the current study at some level the teams contained within could be considered effective in that the missions were accomplished without serious bodily harm. Future research should more explicitly investigate differences between high and low performing teams to more finely delineate areas in which team roles are likely to falter as this could point to potential countermeasures. Moreover, investigation into the temporal dynamics relating to team roles is an area that is wide open. We have begun to provide some initial findings herein as to how time may impact the type of team roles which are enacted. However, future research could begin to examine how often the informally defined team roles examined herein are associated with team enactment of action and transition phases during performance episodes. Leveraging work by Marks et al. (2001), one could imagine that the enactment of particular team roles could be used to drive the efficiency and effectiveness of the phases of cyclical activity which comprise performance episodes. Additionally, future research could begin to highlight those roles that are essential to move teams along different phases of the developmental continuum.

Up to this point, team roles and many other team factors have tended to primarily been examined at a single point in time (usually at the end of the mission), with little attention paid to how the myriad of temporal factors present may impact how they evolve and change with regard to their implementation or instrumentality. It is our hope that the findings presented here and the many new questions that emerge will serve to spur future research in this area.

## Author Contributions

All authors contributed to the writing and assisted in the theoretical development of the manuscript. CB was responsible for conceptualization of the manuscript.

## Conflict of Interest Statement

The authors declare that the research was conducted in the absence of any commercial or financial relationships that could be construed as a potential conflict of interest.
